# Investigating the Osteoregenerative Properties of *Juglans regia* L. Extract on Mesenchymal Stem Cells and Osteoblasts Through Evaluation of Bone Markers: A Pilot Study

**DOI:** 10.3390/jfb16070268

**Published:** 2025-07-21

**Authors:** Alina Hanga-Fărcaș, Gabriela Adriana Filip, Simona Valeria Clichici, Laura Grațiela Vicaș, Olga Şoritău, Otilia Andercou, Luminița Fritea, Mariana Eugenia Mureșan

**Affiliations:** 1 Doctoral School of Biomedical Sciences, University of Oradea, 1 University Street, 410087 Oradea, Romania; alina.hanga@didactic.uoradea.ro; 2 Department of Physiology, Iuliu Hatieganu University of Medicine and Pharmacy, 8 Victor Babes Street, 400347 Cluj-Napoca, Romania; gabriela.filip@umfcluj.ro (G.A.F.); sclichici@umfcluj.ro (S.V.C.); 3 Department of Pharmacy, Faculty of Medicine and Pharmacy, University of Oradea, 10, 1 December Square, 410073 Oradea, Romania; 4 Radiobiology and Tumor Biology Laboratory, Oncological Institute “Prof. Dr. I. Chiricuţă”, 34–36 Republicii Str., 400015 Cluj-Napoca, Romania; otiliabarbos2006@yahoo.com; 5 Department of Preclinical Discipline, Faculty of Medicine and Pharmacy, University of Oradea, 10, 1 December Square, 410073 Oradea, Romania; lfritea@uoradea.ro (L.F.); mmuresan@uoradea.ro (M.E.M.)

**Keywords:** osteoblast, mesenchymal stem cells, bone regeneration, bone biomarkers

## Abstract

Bone tissue regeneration is a complex process that takes place at the level of osteoblasts derived from mesenchymal cells and occurs under the action of multiple signaling pathways and through the expression of osteoregenerative markers. The leaf extract of *Juglans regia* L. (JR) is rich in polyphenols with demonstrated osteoregeneration effects. In the present study, we investigated the extract’s effects on three types of cells with various stages of differentiation: adult mesenchymal stem cells (MSCs), osteoblasts at low passage (O6) and osteoblasts at advanced passage (O10). To assess the efficacy of the walnut leaf extract, in vitro treatments were performed in comparison with ellagic acid (EA) and catechin (CAT). The osteoregenerative properties of the leaf extract were evaluated in terms of cell viability, bone mineralization (by staining with alizarin red) and the expression of osteogenesis markers such as osteocalcin (OC), osteopontin (OPN), dentin matrix acidic phosphoprotein 1 (DMP1) and collagen type 1A. Another compound implicated in oxidative stress response, but also a bone homeostasis regulator, nuclear factor erythroid 2-related factor 2 (NRF2), was studied by immunocytochemistry. Together with collagen amount, alkaline phosphatase (ALP) activity and NF-kB levels were measured in cell lysates and supernatants. The obtained results demonstrate that JR treatment induced osteogenic differentiation and bone mineralization, and it showed protective effects against oxidative stress.

## 1. Introduction

Fractures, bone infections, primary bone tumors and bone metastasis, and osteoporosis are bone lesions with a great health impact and high socioeconomic expenses. Repairing bone injuries with new bone formation is a process involving the activity of osteoprogenitor cells and osteoclasts, accompanied by a dynamic inflammatory response [1].

Bone is composed of a specialized cell network made up of osteocytes, osteoblasts, osteoclasts, MSCs, hematopoietic cells (HSCs) and immune cells, which are in continuous communication and collaboration. At the center of this network are osteocytes, found inside the bone matrix and constantly communicating with the osteoblasts and osteoclasts on the surface, providing a well-proportioned remodeling process through signaling pathways. MSCs differentiate into osteoblasts under the influence of some molecules such as hormones, cytokines and various growth factors, representing potential for bone regeneration. Immune cells also play an active role; there is an equilibrium between proinflammatory and anti-inflammatory signals, affecting bone metabolism [2].

During the process of proliferation and differentiation of osteoblasts from MSCs, they secrete different proteins—growth factors, transcription factors and enzymes—each of them with distinct periods of expression during osteoblast maturation. Some of the most important expression markers are Runx2, ALP, collagen type I (Col-1), OC, OPN and transforming growth factor-β (TGF-β) [3].

The process of osteoblastogenesis in vitro from MSCs can be divided into three overlapping steps, with the expression of two transcription factors being mandatory: Runt-related transcription factor 2 (Runx2) and osterix (OSX). In the first phase, MSCs undergo proliferation and the expressions of collagen 1 and OPN are amplified. The second phase of matrix maturation occurs when proliferation begins to decrease. In this phase, collagen 1 is still produced and secreted in the extracellular space, and ALP activity is significantly amplified. OSX expression also increases, being essential in the ossification process, and Runx2 expression decreases because it inhibits mineralization. The last phase is mineralization, in which collagenase expression reaches its maximum, being necessary in the process of remodeling the newly formed bone matrix [4]. Runx2 is also necessary for non-collagenous protein expression such as OC and bone sialoprotein (BSP). Their expression increases with pre-osteoblast transformation into fully differentiated osteoblasts. BSP binds to collagen I, resulting in hydroxyapatite crystals’ formation and initiating bone mineralization, while OC determines the binding of calcium and hydroxyapatite. Osteoblasts surround themselves with their own matrix, forming an osteoid. Later, osteoblasts differentiate into osteocytes or sometimes they can line cells or undergo apoptosis [5].

Osteopontin (OPN), a non-collagen phosphoprotein mainly secreted by osteoblasts, has a role in bone metabolism and hemostasis. OPN is involved in the proliferation, migration and adhesion of bone-related cells [6]. Produced by osteocytes, dentin matrix protein (DMP1) is an acidic phosphorylated extracellular matrix non-collagenous bone protein involved in the mineralization of the bone matrix by binding to Ca^2+^ molecules [7]. DMP1 is a significant mineralization regulator and thereby a regulator of responses mediated by osteocytes to mechanical load [8]. Another important player in regulating bone homeostasis is nuclear factor erythroid 2-related factor 2 (NRF2). NRF2 agonists have protective effects on osteoblasts, osteocytes and stem cells, while osteoclast differentiation is inhibited by resistance to oxidative stress [9]. The process of bone remodeling is continuous and takes place throughout life, representing an equilibrium between processes of bone resorption carried out by osteoclasts and processes of bone formation performed by osteoblasts. This balance can be disrupted by inflammation and oxidative stress, which facilitate osteoclastogenesis and inhibit osteoblastogenesis. In inflammation, cytokines have an important role, disrupting normal remodeling, separating the two processes of bone resorption and bone formation [10]. The transcription factor nuclear factor kappa B (NF-kB) is a regulator of cellular processes, inflammatory processes and the immune response activated by proinflammatory cytokines: tumor necrosis factor (TNF) and interleukin-17. It also represents a significant contribution in the differentiation processes of stem cells and PDLSCs (human periodontal ligament tissue-derived MSCs) [11,12,13,14].

The most common bone disease, osteoporosis, is a complex pathology whose main mechanism is the bone resorption/bone formation disequilibrium. This is why research is focused on finding effective remedies to treat this condition. The menopausal status is the most well-known cause of osteoporosis, where low levels of estrogen interrupt bone remodeling. Estrogen binds to estrogen receptors at the level of osteoblasts and favors the synthesis of osteoprotegerin (OPG), the “decoy receptor” of the receptor activator of the NF-κB ligand (RANKL) [15]. Low estrogen levels will alter the estrogen target genes’ expression, which will induce an increase in the secretion of TNF and interleukins IL-1, IL-6 and IL-1β. Also, due to the low level of OPG, RANKL will bind to the receptor activator of NF-κB (RANK) on osteoclasts and will promote their maturation and bone resorption [16]. Osteoporosis in allopathic medicine is commonly treated with hormone therapies like estrogen or testosterone replacement, or with inhibitors of osteoclasts (bisphosphonates). Severe osteoporosis with high risk of fractures is treated with parathyroid hormone analogs. These treatments, although they have achieved certain effects in clinical treatment, besides being quite expensive, can be associated with some unwanted adverse effects, one of them being osteonecrosis of the jaw [17]. For this reason, numerous studies have focused on traditional medicine, aiming toward osteoporosis prevention and treatment without severe complications. Various phytochemical compounds extracted from several plants present huge potential in the bone regeneration process because of their pro-estrogenic activity, antioxidant capacity, anti-inflammatory property and modulation of pathways for bone signaling [18,19,20]. Many approaches to osteoporosis treatment and prevention with phyto-biocompounds are presented in the literature. The obtained effects are induced by certain chemically active ingredients from plants (secondary metabolites) like resveratrol, naringin and ginsenoside. Traditional Chinese medicine describes such herbs and their extracts with proven effects in in vivo and in vitro studies on osteoporosis [21].

*Juglans regia* L. (JR), or walnut, is known to possess anti-inflammatory and antioxidant activities. All the plant’s parts are used in traditional medicine, not only for its nutritional properties, but also as a curative choice in a great diversity of diseases: fungal, viral and bacterial infections, oncological diseases, type 2 diabetes and pathologies associated with arterial hypertension [22]. Walnut leaves are employed in natural medicine especially for treating some pathological conditions such as skin ulcers, venous insufficiency, hyperhidrosis, skin irritation and hemorrhoidal disease [23]. Until now, walnut leaves’ osteoregenerative effects have not been studied extensively. According to Pang X. et al., JR leaves exert a positive effect on bone metabolism, particularly on osteogenic gene expression in human bone marrow MSCs [24]. In our previous work, we have proved the relevant antioxidant effect and bone protective activity of JR leaf extract, as well as the accumulation of Ca^2+^ and phosphate (PO_4_^3−^) in the bone structure in a rat model [25]. 

**Scheme 1:**
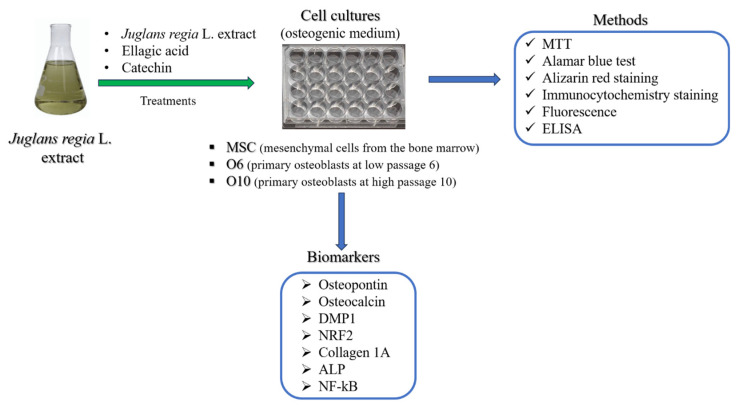
The experimental design of this study.

The objective of the present study was to assess the potential of JR leaf extract in bone regeneration by assessing the effect on MSCs from bone marrow and primary osteoblasts in two stages of differentiation (pre-osteoblast and osteoblast) exposed to osteogenic medium (Scheme 1). In order to delineate the effects of EA and CAT, two biocompounds contained in the JR leaf extract in different proportions, JR’s effect was compared with EA and CAT treatments.

## 2. Materials and Methods

### 2.1. Cell Cultures

Three primary cell lines were used: adult MSCs from bone marrow, primary osteoblasts at low passage (passage 6-called O6) and primary osteoblasts at high passage (O10).

Human osteoblasts were purchased from a human patient with the approval of the Ethics Council of the County Emergency Clinical Hospital of Oradea, Romania (no. 7700/02.03.2023). Bone fragments were harvested in aseptic conditions from a comminuted femoral neck fracture from a patient aged 77 years old. The bone fragments were immersed in transport medium (DMEM/F12 with 10% bovine serum) and were processed within 24 h of harvesting by mechanical fragmentation, obtaining bone pieces with sizes of 2 × 2 mm. An enzymatic digestion step with collagenase IV 1 mg mL^−1^ was applied for 2 h, followed by inactivation of enzyme activity with medium containing 10% FBS. Several washes of fragments were performed with PBS (phosphate-buffered saline) and complete medium. The cells detached during mechanical and enzymatic processing were collected, and supernatants were filtered with 70 nm Filcons meshes (Dako). The obtained cell suspension and bone fragments were centrifuged at 1200 rpm for 10 min, and the pellets were resuspended in complete standard medium consisting of DMEM high glucose/F12 without phenol red 15% fetal calf serum (FCS), 1% non-essential amino acids (NEAs), 1% antibiotic (penicillin–streptomycin) and 2 mM L^−1^ glutamine. All reagents were purchased from Sigma-Aldrich (St Louis, MO, USA). After 14 days, when fibroblastoid cells migrated around the bone fragments, the first passage was performed. The cells showed a proliferation rate that determined the passage to be performed at 3–5 days. In our study, cells at two different passages were used, passage 6 and passage 10, which under the influence of the osteogenic differentiation medium showed distinct morphologies. Osteogenic medium (OS medium) was composed of DMEM high glucose/F12 without phenol red 15% FCS, 1% NEA, 1% antibiotic (penicillin–streptomycin), 2 mM L^−1^ glutamine, ascorbic acid phosphate (50 µg mL^−1^), dexamethasone (20 µM) and β-glycerophosphate (10 mM).

Adults MSCs derived from human bone marrow from the iliac crest, isolated and characterized in a previous study by our team [26], were obtained from an orthopedic patient during a total hip replacement, with the patient giving informed consent and the ethical legal laws being adhered to. The cells were thawed from a batch stored in liquid nitrogen and expanded using standard stem cell medium consisting of DMEM with 4.5 g glucose/F12-HAM (1/1) with 15% FBS (fetal bovine serum), 2 mM L^−1^ glutamine, 1% penicillin–streptomycin solution, 1% NEA solution, 55 µM β-mercaptoethanol and 1 mM sodium pyruvate. All reagents used in cell cultures were provided by Sigma-Adrich. Isolated MSCs were positive for surface pluripotent stem cell markers Nanog, Oct ¾, SOX2, SSEA-4, CD29 and CD105 [26]. The experimental protocol was approved by the Ethical Committee of “Iuliu Hatieganu” University (16 September November 2021).

Treatment experiments were performed with the walnut extract (JR), EA and CAT aqueous solutions, previously filtered with 0.22 nm filters, with the purpose of sterilizing the solutions. CAT was solubilized in double-distilled water to obtain a stock solution of 3.22 mg mL^−1^. The CAT working concentration was 0.32 µg mL^−1^, corresponding to the quantification of the contained CAT concentration in walnut extracts. The EA solution had a concentration of 1%. The 10% solution of walnut extract was obtained as presented in our previous study [25].

### 2.2. Establishing the Working Doses of Walnut Extract (JR), EA and CAT

The cytotoxicity levels of JR extract, EA and CAT were investigated by using the MTT assay. MTT is a tetrazolium salt (3-[4,5-dimethylthiazol-2-yl]-2,5-diphenyl tetrazolium bromide) used to highlight viable cells. MTT solution dissolved in saline buffers without phenol red is yellow. Mitochondrial dehydrogenases of viable cells cleave the tetrazolium ring, producing purple formazan crystals that are insoluble in aqueous solutions. The crystals are dissolved in dimethyl sulfoxide (DMSO) or in acidified isopropanol, resulting in a purple solution whose optical density can be measured spectrophotometrically. An increase or decrease in cell numbers also produces a concomitant change in the amount of formazan formed, indicating the degree of cytotoxicity caused by the test material. Adult MSCs from the bone marrow and osteoblastic cells in two stages of differentiation (pre-osteoblast—O6 and osteoblast—O10) were seeded on flat-bottom 96-well plates from Nunclon (Nunc) at a density of 10^4^ cells in 200 µL standard medium/well. After 24 h, when the cells reached a subconfluence of 60–70%, serial dilutions of JR extract, EA and CAT were used. The determinations were performed in triplicate in two independent experiments. The cells’ viability response to the three compounds was investigated after a longer period of time because in a previous experiment it was observed that after 24 h the effects of the treatments on cell viability and proliferation were minimal. After treatments, the plates were incubated for 72 h. The MTT test consisted of removing the medium from the surface of the cells and adding 100 μL of MTT solution/well (concentration of 1 mg mL^−1^). The plates were then incubated for 1 h in the dark at 37 °C and 5% CO_2_ to allow the MTT to be metabolized by mitochondrial reductases into formazan crystals. The MTT solution was removed and 150 μL DMSO/well was added, homogenizing the samples by using a shaker at 37 °C for 5 min. The resulting purple formazan was dissolved, and optical densities were determined at 570 nm using a BioTek Synergy 2 microplate reader (Winooski, VT, USA).

### 2.3. The alamarBlue Viability Test on Long-Term Cultures Treated with JR Extract, EA and CAT

The alamarBlue assay was employed for the viability determination of the three cell lines cultivated over a longer period (the analysis was performed at 3 and 6 days). The two treatments were performed at 24 h and 72 h, inducing osteogenic differentiation with OS medium. Cells were seeded on 96-well plates with a flat bottom at 10^4^ cells in 200 µL OS differentiation medium/well. After 24 h, when the cells reached a subconfluence of 60–70%, the treatments with walnut extract, EA and CAT were administered at the same dilution x26.6 with the following concentrations: JR extract (0.5 mg mL^−1^), EA (0.05 mg mL^−1^) and CAT (0.15 µg mL^−1^). At 72 h and 6 days, the medium was extracted from the wells and 100 µL of medium with 10% alamarBlue was added. After one hour of incubation at 37 °C and 5% CO_2_ in the dark, the plates were analyzed with the Biotek Synergy 2 fluorescence plate reader using the 560/590 nm (excitation/emission) filter. Statistical analysis was performed with the two-way ANOVA program, comparing the values at 3 days with those at 6 days for each treatment and the control at each time interval with the values obtained in the treated cells.

### 2.4. Evaluation of Mineralization by Alizarin Red Staining

Alizarin red staining was performed for all three cell lines to observe the differences in behavior between the different stages of differentiation of osteoblastic cells: MSC, O6 and O10. They were seeded in plates with 24 wells with a cell density of 5 × 10^4^ cells/well in 1 mL/well of OS differentiation medium. The cells were cultured for 14 days, and 4 treatments were performed with the same dose of JR, EA and CAT (dilution × 26.6). At the end of cultivation, the cells were fixed with 4% paraformaldehyde for 20 min and washed three times with PBS, then with double-distilled water. Exposure of 20 min to 2% alizarin red solution (pH 4.2) was applied with gentle agitation on an orbital shaker. The staining solution was discarded and extensive washes with double-distilled water were performed, with the last wash using PBS. Microscopic images were taken to visualize the calcium deposits under an inverted phase microscope, Zeiss Axiovert D1, using an AxioCAM MRC color camera. Quantification of mineralization was performed with a 10% cetylpyridinium chloride (CPC) solution, a detergent that solubilizes calcium deposits (violet coloration that can be read on a microplate reader at 562 nm). PBS was extracted from the wells, and 10% CPC solution was added, with 15 min incubation on an orbital shaker. Aliquots were extracted into a 96-well plate and readings were performed on a Biotek Synergy2 reader at a wavelength of 562 nm.

### 2.5. Evaluation of Osteogenic Differentiation by Immunocytochemical Staining

MSC, O6 and O10 cells were seeded on 16-well Nunc chamber slides and cultured in osteoinductive medium for 10 days with two treatments each of JR extract (0.5 mg mL^−1^), EA (0.05 mg mL^−1^) and CAT (0.15 µg mL^−1^). Cells were fixed with 4% paraformaldehyde (20 min), then permeabilized with 0.02% Triton X100 solution for 15 min. Non-specific fixation was blocked with 10% bovine albumin for 15 min. Exposure to primary monoclonal antibodies was performed overnight at 4 °C. The monoclonal antibodies used in this experiment were anti-human mouse IgG1 (Invitrogen-Thermo Fisher Scientific, Waltham, MA, USA), anti-human mouse IgG2a osteocalcin (Santa Cruz Biotechnologies, Heidelberg, Germany), anti-human mouse monoclonal IgG1 collagen 1a (Santa Cruz Biotechnologies), anti-human DMP1 (Invitrogen) and Anti-NRF2 polyclonal rabbit antibody (ThermoFischer Scientific) (1:100 dilution).

Secondary antibodies labeled with FITC (Santa Cruz Biotechnogies, Heidelberg, Germany) were applied to the samples using the same dilution as the corresponding primary antibody for 45 min at room temperature in the dark. The dilution ratio of primary and secondary antibodies was 1:50, except for the Anti-NRF2 antibody where the dilution was 1:100. Nuclei staining was performed with a mounting medium containing DAPI. Fluorescence images were taken with a Nikon Elipse E600 fluorescence microscope using a color digital camera under the same exposure conditions at different wavelengths (346 nm, 488 nm).

The mean green fluorescence intensity (represented as arbitrary units) of the captured OPN and OC images were normalized to nuclei number using ImageJ (v1.52P, NIH).

### 2.6. Determination of Collagen Levels from Supernatants and Cell Lysates

For this study, MSC, O6 and O10 cells were seeded in 6-well plates at a cell density of 2 × 10^5^ cells/well in 2 mL of osteogenic medium. Three treatments with JR extract (0.5 mg mL^−1^), EA (0.05 mg mL^−1^) and CAT (0.15 µg mL^−1^) were performed. Supernatants were collected on days 0, 3, 7 and 10. Finally, on day 10, cell lysis was performed using CelLytic™ MT Cell Lysis Reagent (Sigma Aldrich Chemicals GmbH, St Louis, MO, USA). All collected samples were kept in a freezer at −80 °C until analysis. The Collagen Assay Kit (Sigma Aldrich), a simple and sensitive method in fluorescence, was used for collagen dosage. In the first step of this procedure, the collagen in the samples was enzymatically digested into peptides under incubation for 60 min at 37 °C. The obtained N-terminal glycine-containing peptides subsequently reacted with the dye reagent to form a fluorescent complex for 10 min at 37 °C. The fluorescence intensity of the samples, directly proportional to the collagen concentration, was measured at 375/465 nm using a microplate reader under fluorescence at λex = 375/λem = 465 nm, Biotek Synergy2. Extrapolation of the obtained results was carried out from the standard curve using GraphPad Prism 5 software, obtaining collagen concentrations in µg mL^−1^. Comparison between control values at time 0 (T0) and the obtained values at different points of time (3, 7 and 10 days) was performed with one-way analysis of variance followed by the “Bonferroni post test”.

### 2.7. Assessment of ALP Activity

The effects of JR, EA and CAT were evaluated in terms of ALP activity in culture medium supernatants and from cell lysates of MSC, O6 and O10 cells. The cultivation conditions and treatments were the same as those described for collagen dosing, with cultivation of cells in osteogenic medium and three treatments with JR extract (0.5 mg mL^−1^), EA (0.05 mg mL^−1^) and CAT (0.15 µg mL^−1^). Supernatants were collected on days 0, 7 and 10 and cell lysates on day 10.

The activity of ALP was assessed by using the Alkaline Phosphatase Detection Kit, Fluorescence (Sigma Aldrich), whose principle is based on the hydrolyzation of ALP from samples of p-nitrophenyl phosphate into a yellow-colored product with absorbance at 405 nm. The rate of reaction is directly proportional to the enzyme activity. The samples were thawed at room temperature. In a plate with 96 wells, 20 µL of each sample was added in duplicate, using as a negative control medium DMEM/F-12 without phenol red and as a positive control different concentrations of the control enzyme. The 96-well plate was incubated at 65 °C for 10 min to minimize background enzyme activity, followed by sudden cooling on ice for 2 min. The substrate solution at a concentration of 10 mM (disodium salt of 4-methylumbelliferyl phosphate) was added to the wells containing 20 µL of dilution buffer and 160 µL of fluorescent assay buffer. After a short agitation, fluorescence intensity readings were taken on the Biotek Synergy 2 microplate fluorescence reader. The fluorometer was set to 360 nm excitation and 440 nm emission. The results were processed statistically using the analysis software GraphPad Prism 5 and one-way analysis of variance followed by the “Bonferroni post test”.

### 2.8. Evaluation of NFkB p105 Levels from Supernatants and Cell Lysates

The human nuclear factor NF-kappa-B p105 subunit levels were measured by using a sandwich-ELISA method (Elabscience^®^ Human NF-κBp105 ELISA Kit-Houston, Texas, USA) with medium supernatants and cell lysates from MSC, O6 and O10 cells cultivated for 10 days in osteogenic medium with exposure to JR extract (0.5 mg mL^−1^), EA (0.05 mg mL^−1^) and CAT (0.15 µg mL^−1^). Supernatants were collected on days 0, 7 and 10 and cell lysates on day 10. The samples, standards and negative controls were added to the pre-coated plate with a specific antibody for human NF-κBp105 and incubated for 90 min at 37 °C. A biotinylated detection antibody was added to each well with an incubation of 1 h at 37 °C. The optical density was measured spectrophotometrically at a wavelength of 450 nm using the Biotek Synergy 2 microplate reader (Winooski, VT, USA). The concentrations in µg mL^−1^ of human NF-κBp105 protein were extrapolated from the standard curve.

### 2.9. Statistical Analysis

For statistical analysis, we employed the software GraphPad Prism version 5. The data in the graphs are expressed as mean values with their standard deviations. For multiple comparisons among groups, one-way ANOVA or two-way ANOVA was used, followed by the “Bonferroni post test”, with statistical significance set at *p* ≤ 0.05. Using the one-way ANOVA test for normally distributed variables followed by post tests to assess multiple comparisons represents a conservative method that controls the overall error rate by dividing the level of significance by the number of comparisons being made. The Bonferroni post test was used when a large number of pairwise comparisons were analyzed.

## 3. Results

### 3.1. Establishment of Osteoblast Culture

**Figure 1:**
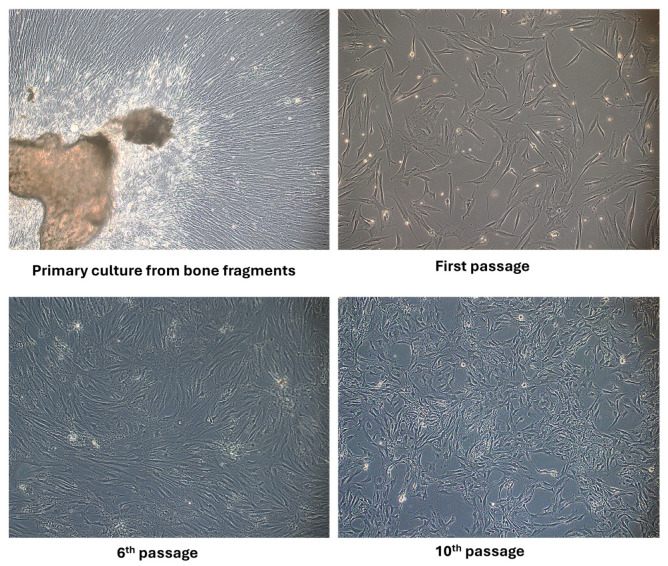
Phase-contrast optical microscopy appearance of osteoblast cells isolated from bone fragments in primary culture, at passage 1, 6 and 10 (objective magnification ×10).

Applying the mechanical and enzymatic processing protocol, the appearance of the first migrated cells to the periphery of the bone fragments was observed in the primary culture after 4–7 days. The first passage was performed after 14 days, when confluence was around 60–70%. After two to three passages, the proliferation rate was increased and a more homogenous fibroblastoid-like shape resembled pre-osteoblasts at passage 6. By applying an osteogenic cultivation medium containing ascorbic acid, β-glycerophosphate and dexamethasone, the cells’ morphology changed, namely smaller dimensions, a more retracted cell body and more prominent nuclei, as seen at passage 10 in Figure 1. The literature describes protocols that also use the method without enzymatic digestion but emphasize certain cultivation conditions. To obtain a pure osteoblast culture, it seems that collagenase treatment is a necessary step to avoid contamination with bone marrow-derived cells. Supplementation of the cultivation medium with ascorbic acid is beneficial for both osteoblast isolation and for collagen’s normal synthesis and secretion by osteoblast cells [27]. Other authors have reported that spontaneous isolation is also possible by defining osteoblasts with specific markers such as Bone Morphogenetic Protein-2,4 collagen type I and ALP activity. They found that the time needed in primary cultures to reach confluence depended on the age of the donors and on the method of cell harvesting, an advantage being aged under 65 years old and using the collagenase isolation method [28].

### 3.2. Determining the Working Doses of JR Extract, EA and CAT by Using the MTT Assay

**Figure 2:**
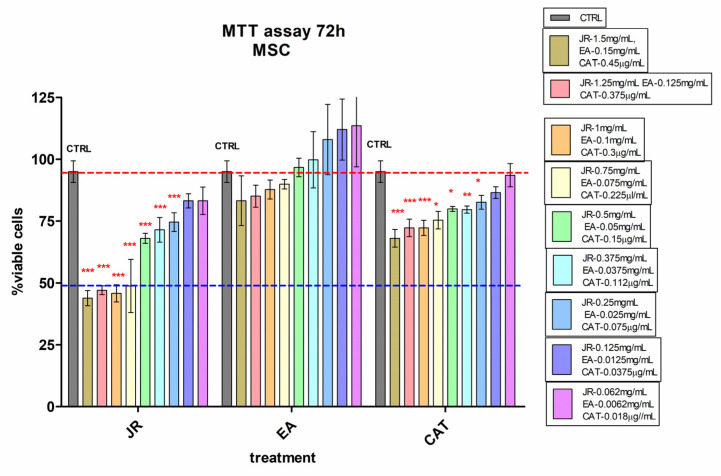
MTT viability test of adult mesenchymal cells (MSCs) from bone marrow, treated for 72 h with walnut leaf extract (JR), ellagic acid (EA) and catechin (CAT). Error bars represent the standard deviations of three separate measurements (*n* = 3). Two-way ANOVA followed by the Bonferroni post test was used for comparison of different treated groups with untreated control cells; * *p* < 0.05, ** *p* < 0.01 and *** *p* < 0.001.

The effects of JR extract, EA and CAT on MSC, O6 and O10 cultured for 72 h in the presence of the treatments were assessed by using the cell viability test, MTT. The obtained results for MSCs are illustrated in Figure 2. High cytotoxicity was observed at doses between 1.5 mg mL^−1^ and 0.75 mg mL^−1^ of JR extract, with a percentage reduction in viable cells below 50% compared to the control untreated cells. The effects of EA were at the level of the untreated control values. CAT induced a cytotoxic effect of about 30% inhibition at higher doses from 1.5 mg mL^−1^ to 0.75 mg mL^−1^ (Figure 2).

**Figure 3:**
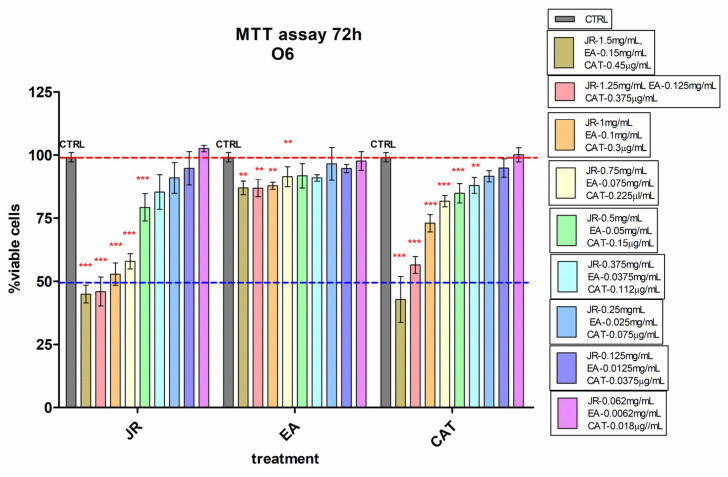
MTT viability test of osteoblastic cells—O6 treated with walnut leaf extract (JR), ellagic acid (EA) and catechin (CAT). Error bars represent the standard deviations of three separate measurements (*n* = 3). Two-way ANOVA followed by the Bonferroni post test was used for comparison of different treated groups with untreated control cells; ** *p* < 0.01 and *** *p* < 0.001.

Young osteoblastic cells (O6) behaved similarly to adult stem cells in terms of their response to JR, showing lower inhibition at high doses compared to MSCs. A 50% decrease in viability compared to control cells was observed at higher doses of 1.5–1.0 mg mL^−1^ of JR. Instead, they presented increased sensitivity to high concentrations of CAT (Figure 3).

**Figure 4:**
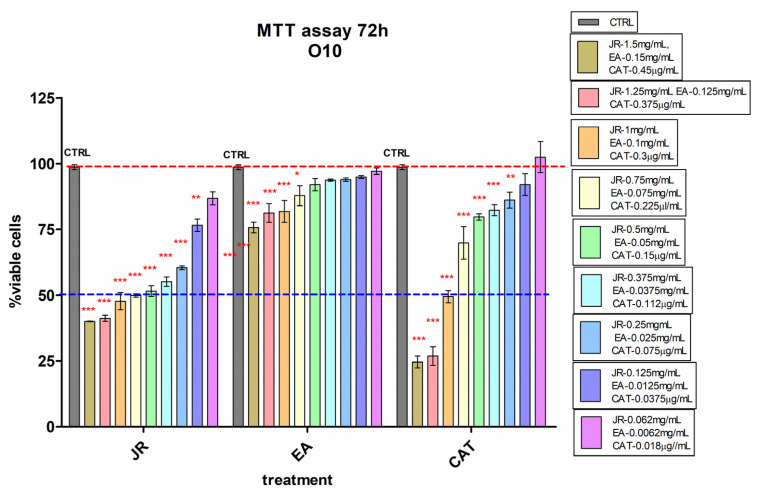
MTT viability test of differentiated osteoblastic cells (O10) treated with walnut leaf extract (JR), ellagic acid (EA) and catechin (CAT). Error bars represent the standard deviations of three separate measurements (*n* = 3). Two-way ANOVA followed by the Bonferroni post test was used for comparison of different treated groups with untreated control cells; * *p* < 0.05, ** *p* < 0.01 and *** *p* < 0.001.

Similar behavior was also observed in O10, but with higher sensitivity even at lower doses of JR up to 0.25 mg mL^−1^ and with a greatly increased cytotoxic response to high doses of CAT (Figure 4).

### 3.3. Long-Term Effects of JR Extract, EA and CAT on Pre-Differentiated Cells’ Viability and Proliferation

The results obtained in the MTT viability test enabled us to select a common dilution (×26.6) for the three treatments that would not be cytotoxic and that at the same time would exert effects on the osteogenesis process. The used concentrations were as follows: JR—0.5 mg mL^−1^, EA—0.05 mg mL^−1^ and CAT—0.15 µg mL^−1^. In the experiment using the alamarBlue test, this dose on cells cultured for a longer period of up to 6 days was followed with the administration of two treatments at 24 h and 3 days. The rationale for using a single dose for the three treatments derived from the viability assays was the observation of differences in the induction of bone differentiation of the three cell lines at different stages of maturity, in a manner similar to what occurs in a whole organism. This can be considered a limitation of this study, determined by the intention of simplification for the interpretation of the results.

**Figure 5:**
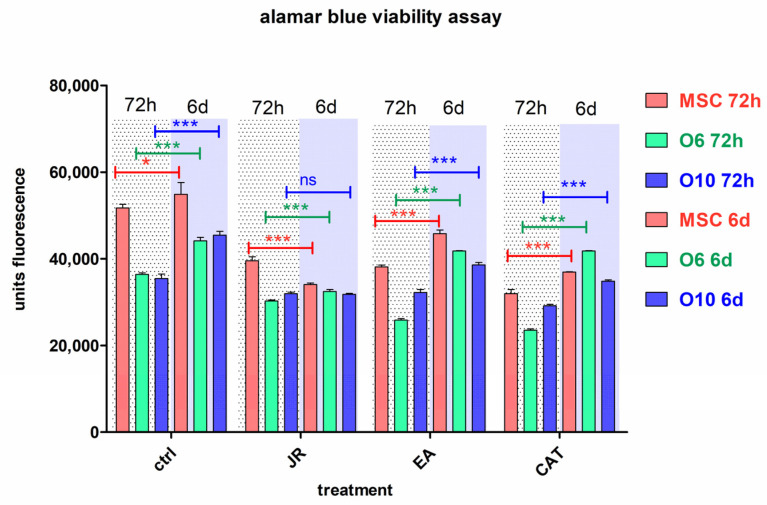
The fluorescence values emitted by cells in the alamarBlue test under the influence of two treatments with JR extract (0.5 mg mL^−1^), EA (0.05 mg mL^−1^) and CAT (0.15 µg mL^−1^). The determinations were made at 3 and 6 days of cultivation after the two treatments. For multiple comparisons for each cell line of controls and treatments responses, two-way ANOVA analysis followed by the Bonferroni post test was performed, estimating the differences between values obtained at 72 h with those at 6 days. Error bars represent the standard deviations of measurements (*n* = 4). ^ns^ *p* > 0.5, * *p* < 0.05 and *** *p* < 0.001.

Statistical analysis with comparison between the values at 3 and 6 days for each treatment (Figure 5) showed that control cells cultivated in osteogenic medium had a higher growth rate in this period, especially osteoblastic cells. All treatments induced a decrease in cell number after 3 days for cells with an early pre-differentiation phenotype—MSC and O6 cells. Treatment with JR at a dilution of ×26.2 (0.5 mg mL^−1^) induced a decrease in MSC proliferation on day 6, unlike treatment with EA and CAT which supported the growth of these cells. Osteoblast cells were not influenced by JR treatment, but EA and CAT administration induced an increase in cell number (Figure 5).

**Figure 6:**
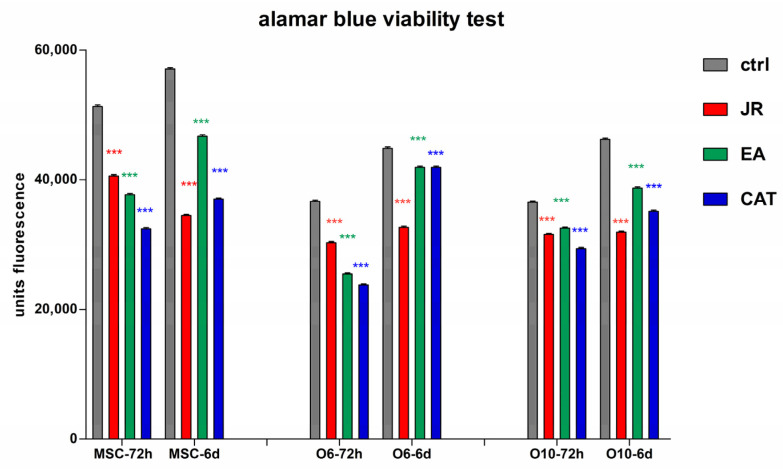
The fluorescence values emitted by cells in the alamarBlue test under the influence of two treatments with JR extract (0.5 mg mL^−1^), EA (0.05 mg mL^−1^) and CAT (0.15 µg mL^−1^). The determinations were made at 3 and 6 days of cultivation after the two treatments. For comparisons of each cell line between controls (blue columns) and treatments responses at the same time of analysis (3 days or 6 days), two-way ANOVA analysis followed by the Bonferroni post test was performed. Error bars represent the standard deviations of measurements (*n* = 4). *** *p* < 0.001.

In another graphic representation of alamarBlue data, the effect at a certain time of the three treatments is revealed. In these graphs, each type of cell is compared with the untreated control and the obtained values after treatments for JR, EA and CAT at the same time (3 days or 6 days). (Figure 6).

An obvious trend in all cell lines was observed in the first time interval of 3 days, where a similar decrease in cell proliferation was noticed for all three treatments, the most pronounced for CAT. After 6 days, the cells treated with EA showed a return to values like those of the untreated cells, especially for the O6 line. Mature osteoblasts O10 showed the lowest degree of variation across all treatments.

### 3.4. Alizarin Red Staining

The differences in behavior between the different stages of osteoblastic cell differentiation were also assessed by alizarin red staining. More visible staining of the differentiated control cells O6 and O10 was noticed compared to the adult MSCs. JR extract and EA induced slightly more distinct mineralization for pre-differentiated cells, while CAT had a cytotoxic effect at this dose.

**Figure 7:**
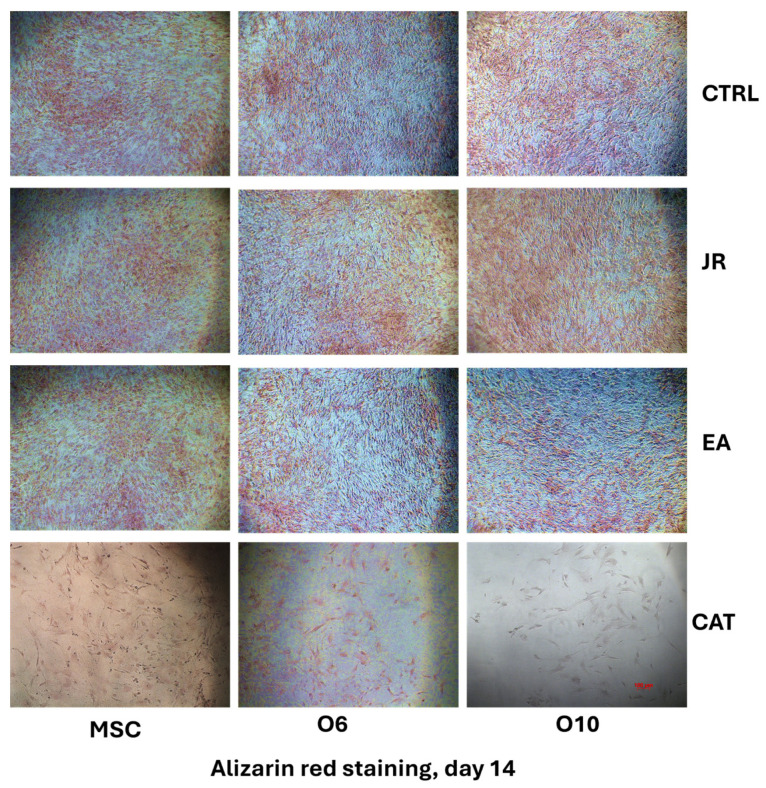
The microscopic appearance of alizarin red staining of MSC and osteoblastic cells after 14 days of cultivation with OS medium and 4 treatments with JR extract (0.5 mg mL^−1^), EA (0.05 mg mL^−1^) and CAT (0.15 µg mL^−1^). (phase-contrast microscopy, ×10 objective). Scale bar = 100 µm.

By using phase-contrast microscopy, it was observed that the mineralization process after 14 days was almost similar between the control cells and those treated with JR, but slightly more pronounced for those treated with EA. CAT greatly decreased the cell viability rate at the dose used (Figure 7).

**Figure 8:**
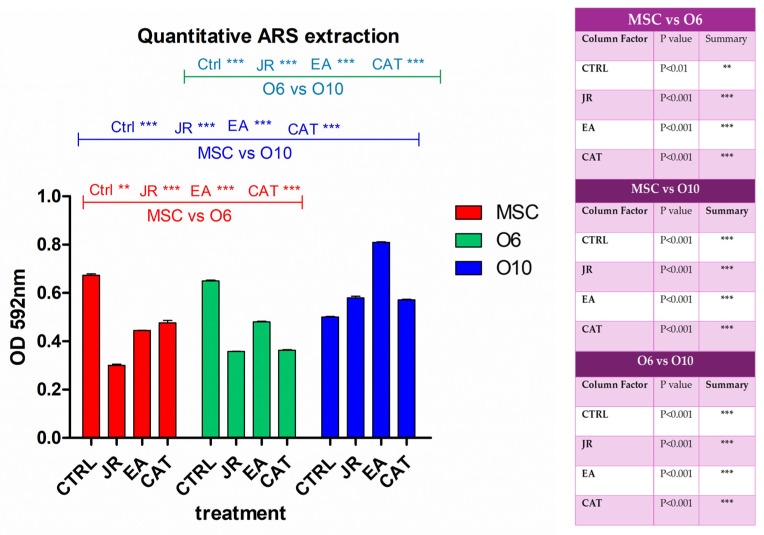
A graphical representation of the results of the quantification of alizarin red staining via extraction with 10% cetylpyridinium chloride. The right panel illustrates the statistical analysis with two-way ANOVA followed by the Bonferroni post test. Experiments: *n* = 3. Values are expressed as the mean ± SD. Error bars represent the standard deviations of measurements. ** *p* < 0.01 and *** *p* < 0.001.

By extracting the alizarin red stain with 10% CPC solution, the degree of mineralization induced by each treatment was quantified according to the degradation of bone differentiation of each cell line. It was observed that JR extract and EA induced the most significant increase in calcium deposition in the most differentiated cells O10, followed by O6 (Figure 8).

### 3.5. Evaluation of Osteogenic Differentiation by Immunocytochemistry

Osteogenic differentiation was evaluated by following the expression of bone markers characteristic of each stage of differentiation. For early differentiation towards osteoblasts, we evaluated OPN and collagen. For the phase of construction and maturation of the bone matrix, we evaluated collagen 1A. For the final phase of mineralization and final differentiation towards osteocytes, we evaluated OC, osteonectine and DMP1.

This study focused on the expression of osteogenesis markers, osteopontin, collagen 1A, osteocalcin and DMP1, depending on the differentiation stage of the three cell lines. In addition, the expression of NRF2 was also monitored, as the NRF2-Keap1 signaling pathway plays a role in cell defense and survival. The transcription factor NRF2 protects cells from toxic substances including xenobiotics and oxidative stress; therefore, NRF2 activators are currently being tested as chemoprotective compounds. The main role of NRF2 is the activation of the cellular antioxidant response by inducing the transcription of a wide range of genes that are able to combat the harmful effects of some extrinsic and intrinsic factors, especially those associated with oxidative stress. Additional tests on the antioxidative response were performed by determining the antioxidant profile of O6 cell lysates (malondialdehyde (MDA) and catalase activity). The obtained data are included in the Supplementary Materials.

**Figure 9:**
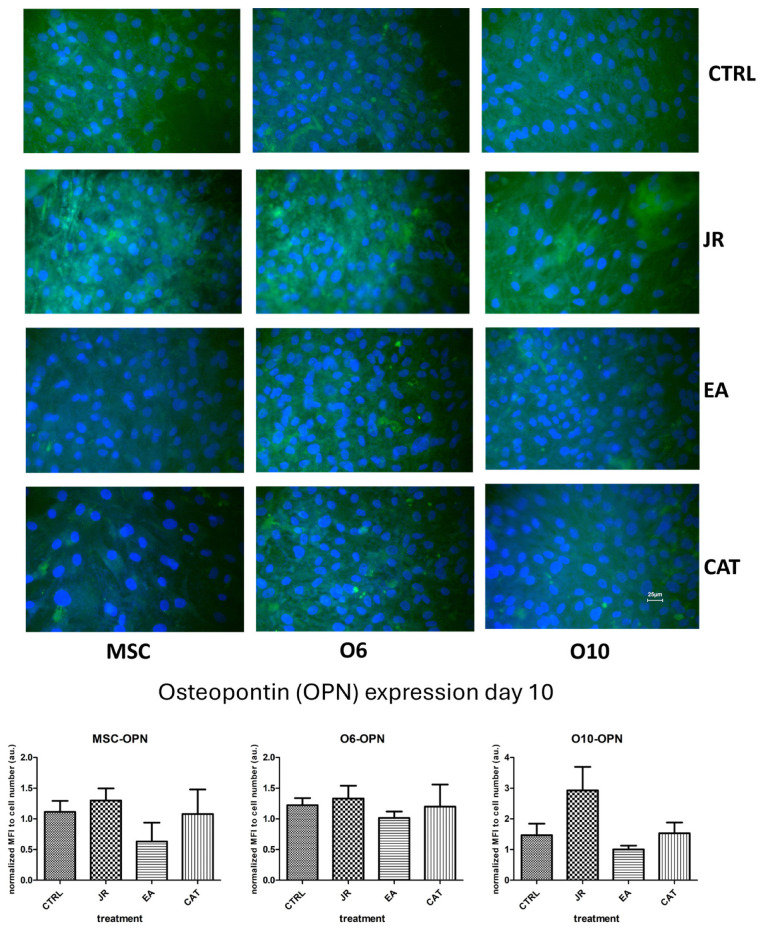
Fluorescence images for osteopontin staining (FITC) of MSC, O6 and O10 cells cultured for 7 days with osteogenic medium and the application of two treatments with JR extract (0.5 mg mL^−1^), EA (0.05 mg mL^−1^) and CAT (0.15 µg mL^−1^). Magnification, ×40 objective. Scale bar = 25 μm. In the bottom panel, graphical representations of the average fluorescence intensity normalized to the number of DAPI-stained nuclei are shown. Data are presented as the mean ± SD (*n* = 3).

The images in Figure 9 reveal several observations. The control cells expressed OPN with a moderate intensity, but the treatment with JR induced an increase in the fluorescence intensity (the highest for the younger cells—MSC and O6). The effect of EA was the opposite, with the increase in OPN expression in cells occurring in a more mature stage of differentiation. Cells exposed to CAT showed levels very similar to control cells.

**Figure 10:**
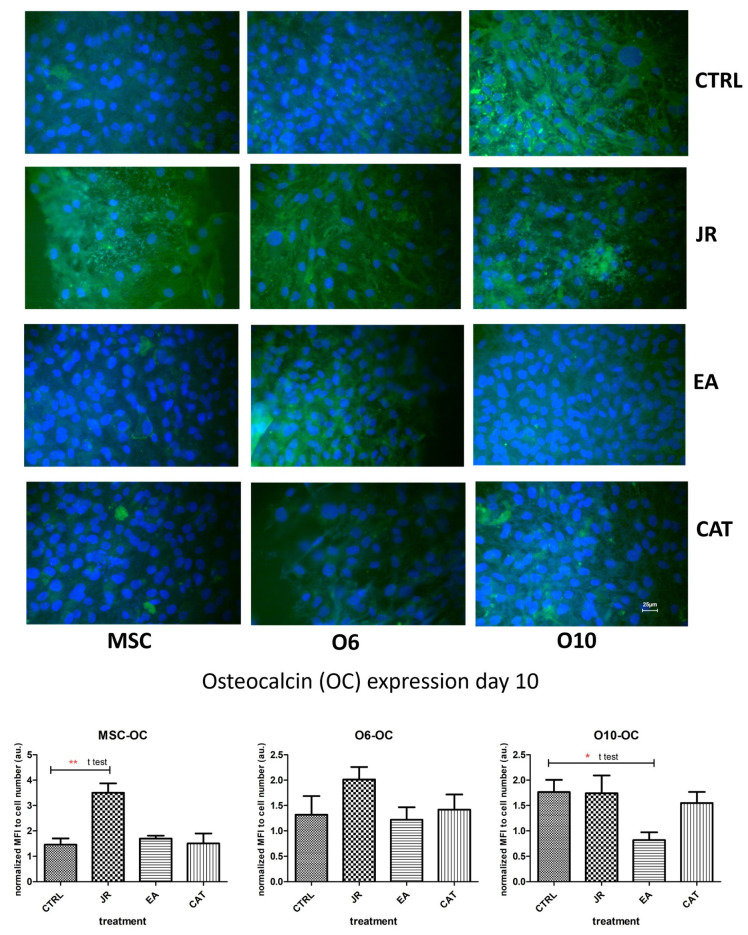
Fluorescence images for osteocalcin staining (FITC) of MSC, O6 and O10 cells cultured for 7 days with osteogenic medium and the application of two treatments with JR extract (0.5 mg mL^−1^), EA (0.05 mg mL^−1^) and CAT (0.15 µg mL^−1^). Magnification, ×40 objective. Scale bar = 25 μm. In the bottom panel, graphical representations of the average fluorescence intensity normalized to the number of DAPI-stained nuclei are shown. Statistics were assessed using a paired *t*-test. Values are expressed as the mean ± SD). * *p* < 0.05, ** *p* < 0.01.

The effect of JR on inducing differentiation towards mature osteoblastic cells was also observed in the staining for the expression of OC. OC is a non-collagen protein that represents approximately 15% of the bone protein matrix. During the process of bone differentiation, OC secretion is very decreased and does not reach maximum levels until the mineralization’s late stages. As shown in Figure 10, the untreated control cells expressed different levels of OC, the most intense being for the most advanced differentiated cells O10. JR extract substantially increased OC expression in MSCs, but also in O6 and O10 osteoblasts. EA augmented the production of OC, especially by younger osteoblasts O6, but also by MSCs, and inhibited OC expression in O10 cells. CAT amplified the expression of OC by MSCs and inhibited its expression by mature osteoblastic cells O6 and O10.

**Figure 11:**
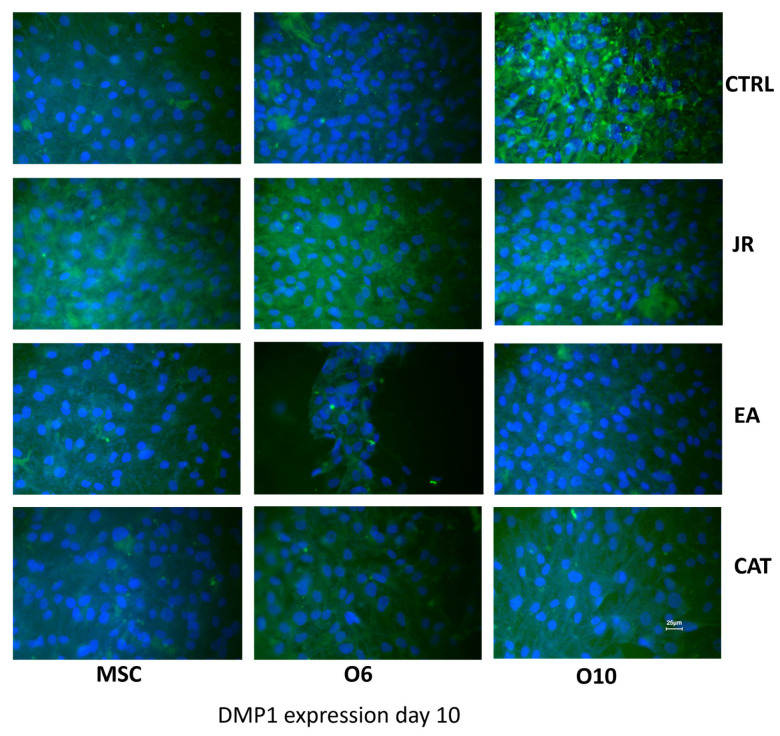
Fluorescence images for DMP1 staining (FITC) of MSC, O6 and O10 cells cultured for 7 days with osteogenic medium and the application of two treatments with JR extract (0.5 mg mL^−1^), EA (0.05 mg mL^−1^) and CAT (0.15 µg mL^−1^). Magnification, ×40 objective. Scale bar = 25 μm.

A similar effect was observed when staining for DMP1 (FITC). DMP1 is a critical protein for the proper mineralization process of bone and dentin; it is present in various cells of bone and dental tissues. In undifferentiated osteoblasts, it is primarily a nuclear protein regulating the expression of osteoblast-specific genes. During osteoblast maturation, the protein becomes phosphorylated and is exported to the extracellular matrix, where it initiates the formation of the mineralized matrix. In Figure 11, the intra-nuclear expression of DMP1 can be seen by young MSCs. This expression was much more intense and localized in the extracellular space for O6 and O10 cells, where the mineralization process was much more advanced. The effect of JR extract increases the synthesis of DMP1 by MSCs, with protein accumulation in the extracellular space. Mature O10 osteoblastic cells presented a slight decrease in DMP1 expression compared to the untreated control. EA acted only on young MSCs and induced a decrease in expression by mature cells. CAT also inhibited the expression of DMP1 in mature osteoblast lines.

**Figure 12:**
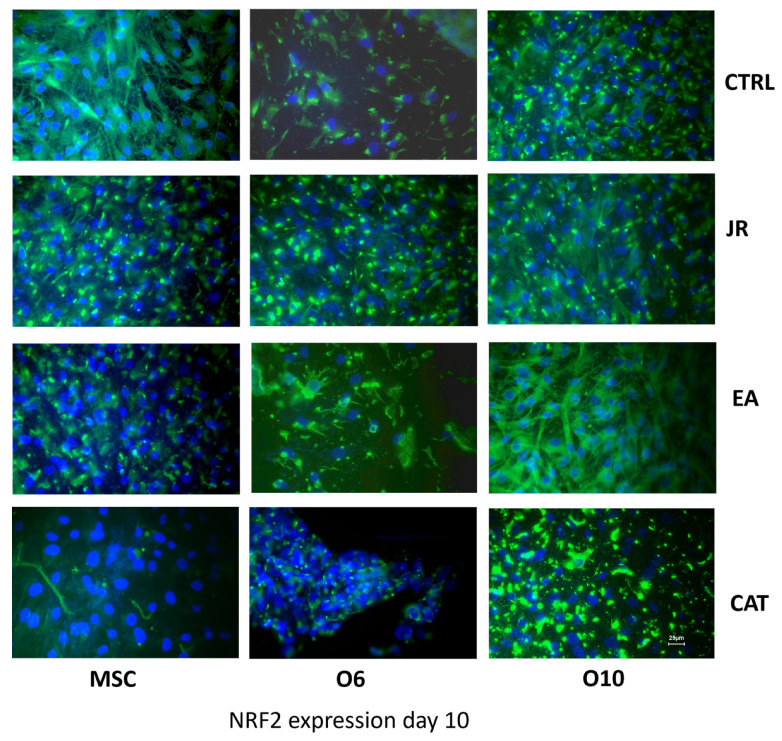
Fluorescence images for NRF2 staining (FITC) of MSC, O6 and O10 cells cultured for 7 days with osteogenic medium and the application of two treatments with JR extract (0.5 mg mL^−1^), EA (0.05 mg mL^−1^) and CAT (0.15 µg mL^−1^). Magnification, ×40 objective. Scale bar = 25 μm.

To evaluate the protection-inducing effects of JR extract, EA and CAT against exogenous factors, especially against oxidative stress, immunocytochemical staining was employed to evaluate the expression of the NRF2 protein/transcription factor. It seems that the stage of osteogenic maturation also determined different expressions of NRF2, with the most intense expression found for mature O10 cells and the lowest level for O6 cells. Both JR extract and EA determined a significant increase in this protein in all three cell lines, meanwhile CAT inhibited the expression of NRF2 by MSCs (Figure 12).

**Figure 13:**
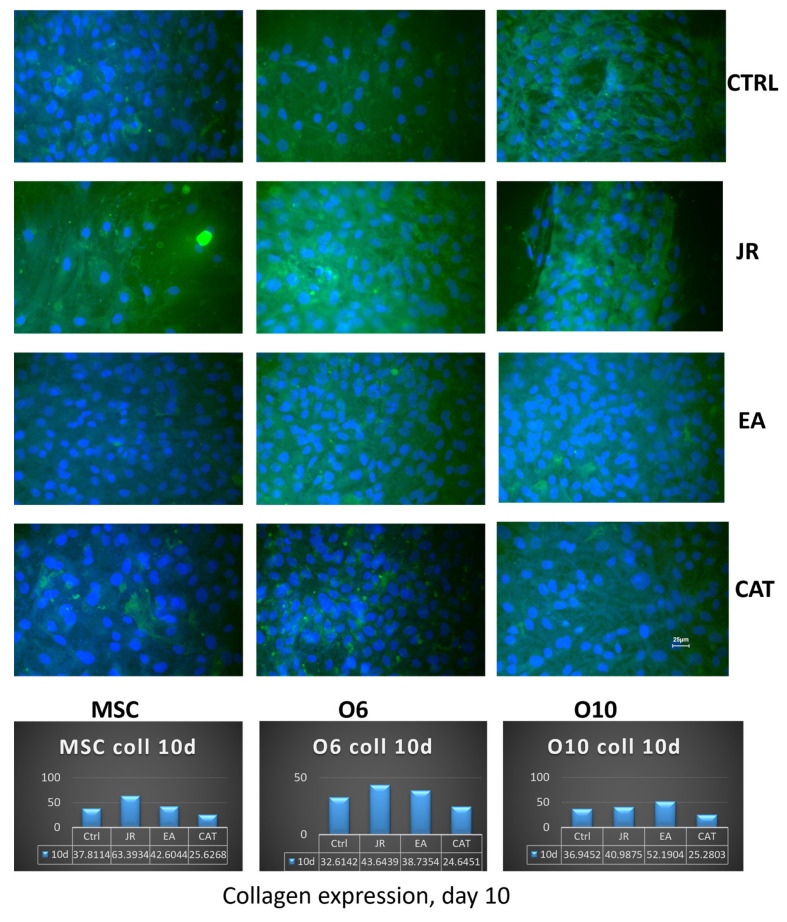
Fluorescence images for collagen 1A staining (FITC) of MSC, O6 and O10 cells cultured for 7 days with osteogenic medium and the application of two treatments with JR extract (0.5 mg mL^−1^), EA (0.05 mg mL^−1^) and CAT (0.15 µg mL^−1^) (×40 objective). Scale bar = 25 μm. The bottom panel corresponds to the collagen measurements (μg mL^−1^) performed after 10 days in the osteogenic cell culture medium.

The expression of collagen 1A was investigated in parallel with collagen dosing from the supernatants after 10 days of cultivation of the three cell lines in osteogenic medium and after the application of three treatments with JR, EA and CAT performed at each medium change. Collagen was expressed intracellularly and partially extracellularly by MSCs, and this expression increased in more mature osteoblasts (O6 and O10). In immunocytochemical staining, this expression was detected in untreated cells, more intense in the case of O10 cells. JR extract increased collagen expression in the supernatants after 10 days of cultivation which were collected from MSC cultures (average 63 μg mL^−1^) and to a lesser extent from O6 (average 43.6 μg mL^−1^) and O10 cells (average 40.9 μg mL^−1^). The effect of EA was demonstrated especially in mature osteoblasts (O10), with increased collagen expression both intracellularly and in the supernatants (average 52 μg mL^−1^). CAT had an effect of increasing intracellular expression only in O6 osteoblasts, but in the supernatants the level decreased for all three cell lines at a level of around 25 μg mL^−1^ (Figure 13).

### 3.6. Collagen Levels in Supernatants and Cell Lysates

**Figure 14:**
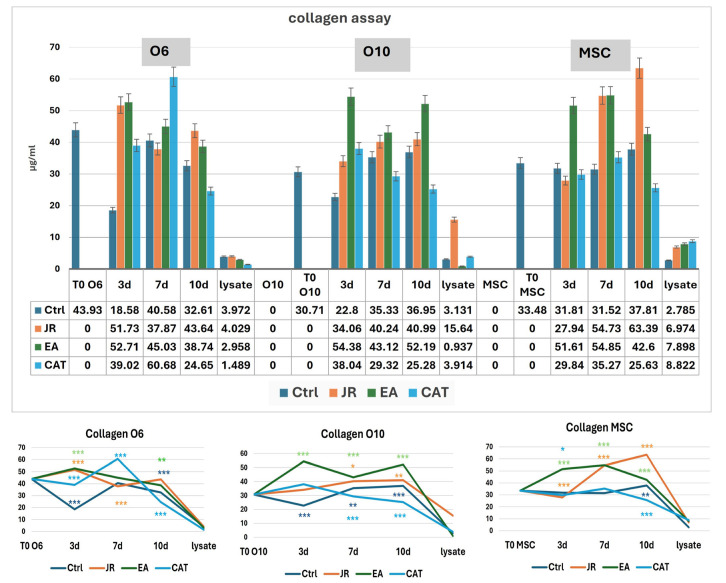
Collagen levels (µg mL^−1^) at time 0 and at 3, 7 and 10 days in the supernatants of MSC, O6 and O10 cell cultures in osteogenic medium and after 3 treatments with JR extract (0.5 mg mL^−1^), EA (0.05 mg mL^−1^) and CAT (0.15 µg mL^−1^). The cellular lysate was harvested 10 days after the culture. The values are expressed in µg mL^−1^. Error bars represent the standard deviations of three measurements (*n* = 3). One-way ANOVA followed by the Bonferroni post test demonstrating the statistically significant effects for each cell line in response to JR, EA and CAT treatments compared to control samples is illustrated in the bottom panel. The *p*-values are set at * *p* < 0.05, ** *p* < 0.01, or *** *p* < 0.001.

For the MSC cell line, we observed a linear evolution of the amount of collagen for the control samples in the first 3–7 days (average 31 μg mL^−1^), with a slight increase to 37 μg mL^−1^ at 10 days. Treatment with JR led to a significant increase in collagen in the culture medium progressively up to 10 days (average 63 µg mL^−1^). EA induced an increase in collagen to values similar to those induced by JR at 7 days (54 μg mL^−1^), while CAT triggered a slight increase in collagen on the 7th day. The younger control osteoblastic cells (O6) showed the highest level of collagen in the medium on day 7. The response to JR and EA was the highest after 3 days, and then the collagen level decreased gradually. CAT induced the maximum increase in collagen on day 7 (60 μg mL^−1^). Mature osteoblastic cells (O10) showed a more moderate response to JR with an increase up to 40 μg mL^−1^ at 7 and 10 days. EA also induced an increase in collagen levels from day 3, while CAT inhibited collagen secretion in O10 cells (Figure 14).

### 3.7. Evaluation of ALP Activity

ALP activity in control samples was the highest in the undifferentiated stem cells, then decreased as MSC differentiation was initiated. Increased ALP activity is described in pluripotent stem cells and related cells as a key marker. Tissue non-specific alkaline phosphatase (TNAP) is an isoenzyme implicated in the initiation of bone mineralization by providing free inorganic phosphate for local hydroxyapatite formation with further deposition in the extracellular space between collagen fibrils. It can also be an inhibitor of the bone matrix via hydrolysis of phosphates [29,30,31].

**Figure 15:**
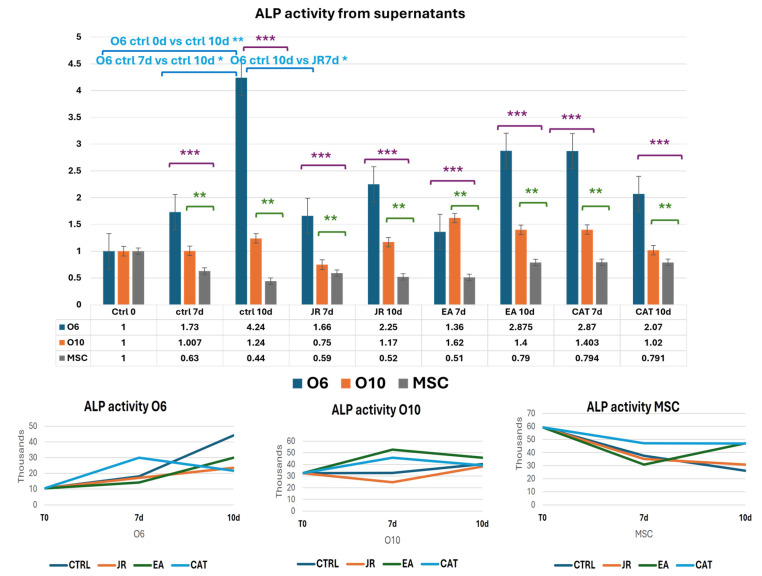
Graphics of induced alkaline phosphatase activity represented as fold-change versus control from supernatants of MSC, O6 and O10 cell cultures in osteogenic medium and after 3 treatments of JR, EA and CAT; samples were collected at time 0, 7 and 10 days. Error bars represent the standard deviations of three measurements (*n* = 3). Statistical analysis representation: One-way ANOVA (Kruskal–Wallis test followed by Dunn’s Multiple Comparison Test) results are illustrated with comparison of cell groups: O6 vs. MSCs (purple brackets and stars *** *p* < 0.001); O10 vs. MSCs (green brackets and stars ** *p* < 0.01). Two-way ANOVA analysis is represented with blue brackets and stars * *p* < 0.05).

**Figure 16:**
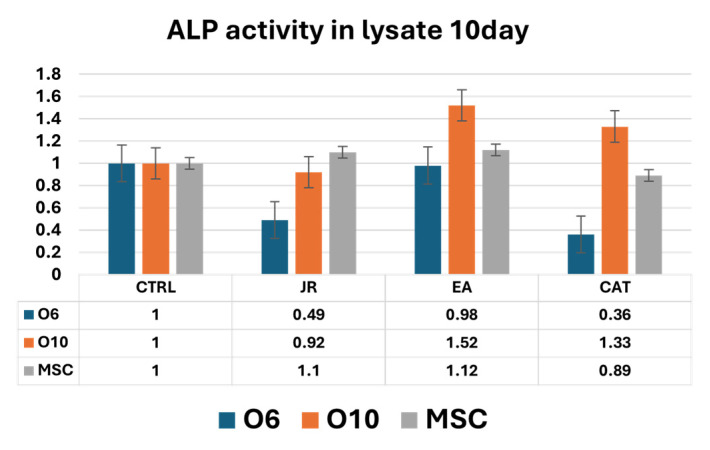
A graphic representation of alkaline phosphatase activity (as fold-change versus control) measured from cell lysate samples of MSC, O6 and O10 cell cultures in osteogenic medium and after 3 treatments of JR, EA and CAT; samples were collected at 10 days. Error bars represent the standard deviations of three measurements (*n* = 3).

Untreated O6 cells had the lowest activity at the initiation of cultures (T0), but ALP activity increased progressively until day 10. Control O10 cells showed relatively increased levels of ALP activity compared to O6, but these levels remained relatively constant throughout the 10 days of culture. JR compared to untreated samples did not induce an increase in ALP activity. It was observed that JR on day 10 was more active on O10 cells. EA was the most active on O10 cells, with an increase in values above those measured in untreated control cells. MSCs also showed increased activity 10 days after EA treatment, above the levels in controls at the same time. CAT also had stimulatory effects on O10 cells and MSCs, especially on day 7 (Figure 15 and Figure 16).

Intracellular ALP activity observed in cell lysates harvested after 10 days of culture was increased in control MSCs compared to O6 and O10 cells. JR induced a decrease in ALP values for O6 cells without affecting those of MSC and O10. EA and CAT induced an increase in ALP activity in O10 and MSCs and a decrease in ALP values for O6 (Figure 16).

### 3.8. Evaluation of NF-kB Levels

**Figure 17:**
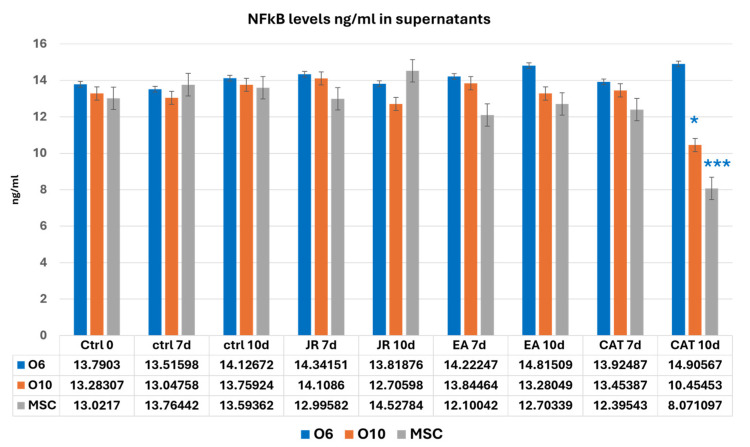
A graphic representation of NF-kB values (ng mL^−1^) evaluated from cell supernatants of MSC, O6 and O10 cell cultures in osteogenic medium and after 3 treatments of JR, EA and CAT; samples were collected at 0, 3 and 10 days. Error bars represent the standard deviations of three measurements (*n* = 3). Two-way ANOVA followed by the Bonferroni post test for evaluation of significant differences between cell lines in response to JR, EA and CAT treatments. The *p*-values are set as * *p* < 0.05 or *** *p* < 0.001.

NF-kB values showed variations without significant differences in almost all the samples analyzed. CAT, through its cytotoxic action, reduced the number of cells to much lower values at 10 days for MSCs and O10 cells. Statistical differences in NF-kB (two-way ANOVA followed by Bonferroni posttest) were only registered for CAT at 10 days of cultivation compared to the control samples (Figure 17).

**Figure 18:**
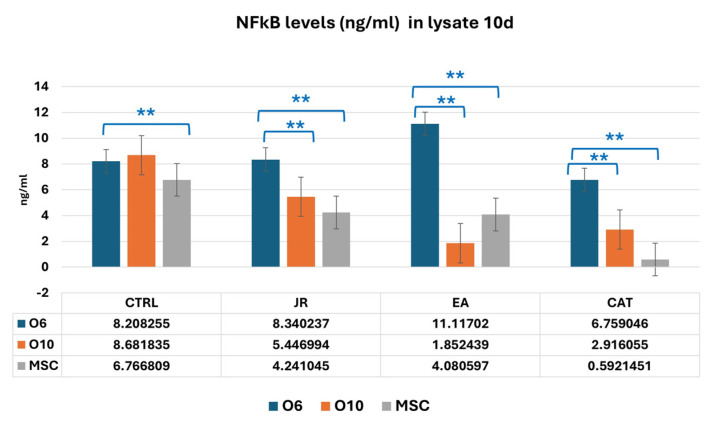
A graphic representation of NF-kB values (ng mL^−1^) evaluated from cell lysate samples of MSC, O6 and O10 cell cultures in osteogenic medium and after 3 treatments of JR, EA and CAT; samples were collected at 10 days. Error bars represent the standard deviations of three measurements (*n* = 3). One-way ANOVA followed by the Bonferroni post test for evaluation of significant differences between cell lines in response to JR, EA and CAT treatments. The *p*-values is set as ** *p* < 0.05.

It was observed, however, that in the lysates harvested at 10 days of culture, that young osteoblastic cells responded with an increase in NF-kB expression to the treatment with EA, in contrast to much lower values obtained in mature osteoblasts and MSCs (Figure 18).

## 4. Discussion

In the present study, we analyzed the effects of treatment with JR extract compared to EA and CAT on cells at different stages of bone differentiation to understand at which stage of bone differentiation these bio-active agents act. This pilot study investigated the treatment response of osteoblasts derived from osteoporotic tissue. The bone tissue was collected from a 77-year-old donor who had a femoral neck fracture due to osteoporosis. These results cannot be extended to the general population (younger or male persons). Therefore, we plan to enlarge our studies by involving multiple donors for future validation.

Our results show a greater sensitivity of mature osteoblasts and to a lesser extent osteoblastic progenitors (MSCs and pre-osteoblasts) to JR, as shown by the MTT viability tests after 72 h of treatment with different doses of JR. In the evaluation of bone differentiation experiments using the OS differentiation medium, the cells were exposed to successive treatments with a predetermined dose as follows: JR extract (0.5 mg mL^−1^), EA (0.05 mg mL^−1^) and CAT (0.15 µg mL^−1^). The rationale for using a single concentration for the three kinds of treatments was to create a comparative parallel with in vivo conditions where the osteoblastic cells are present at different stages of differentiation, so their response to therapy with a predetermined dose will probably determine different responses depending on the cells’ maturity phase. The approach adopted in our experiments represents a limitation that was determined by simplifying the interpretation of the results due to numerous variables. In the experiment in which the cells were exposed to multiple administrations of a non-toxic dose of JR, EA and CAT, it was observed that JR did not induce the proliferation of any of the three cell lines, in contrast to EA and CAT. This observation suggests cytostasis induced by the initiation of a differentiation process often associated with decreased cell proliferation. The mineralization process, assessed with alizarin red staining, was more pronounced for osteoblasts (O6 and O10 cells) treated with EA compared to JR and CAT treatments, the latter leading to an inhibition of calcium deposition.

The intracellular expression of osteogenic markers, collagen, OPN, OC, DMP1 and NRF2 was increased mainly in the young cells (MSC and O6) treated with JR extract. A particularity was the expression of NRF2, which was increased in all cell lines after EA administration. CAT had this effect only on osteoblastic cells. NRF2 is widely present as an inactive form in the cytoplasm of many cell types such as osteoblasts, osteocytes and osteoclasts. NRF2-deficient mice presented a reduced resistance to oxidative stress with increased predisposition to osteoporosis [32]. Oxidative stress exposure determines NRF2 release from its Keap1 inhibitory protein and its translocation into the nucleus, activating various antioxidant and detoxification enzymes. Cytoplasmic expression of NRF2 keeps the bone cells safe from damage induced by oxidative stress, inhibiting the imbalance of bone remodeling and improving fracture healing. Oxidative stress is incriminated to be a well-known risk factor for osteoporosis development. A multitude of natural compounds such as resveratrol, schisandrin A, lutein and psoralen target the NRF2 pathway and consequently reduce osteoporosis prevalence due to oxidative stress regulation [33]. Polyphenolic compounds found in the composition of JR leaf extract, such as caffeic acid, resveratrol, ellagic acid and quercetin, act as indirect antioxidants, increasing NRF2 activity by inducing its target genes [34,35].

**Figure 19:**
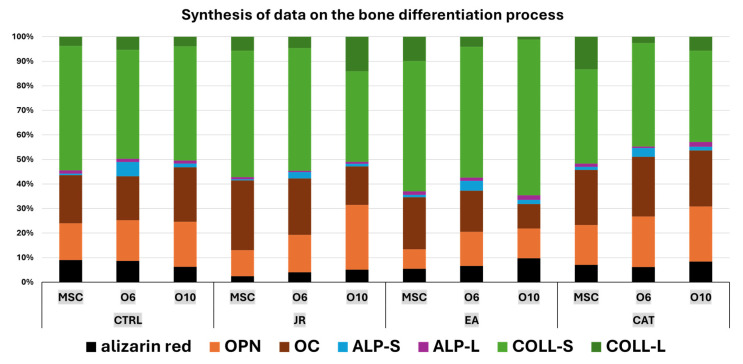
A schematic diagram of the majority of the main elements of bone differentiation with a synthesis of quantifiable results reflecting the response of MSCs and osteoblasts to treatments with JR, EA and CAT (OPN—osteopontin, OC—osteocalcin, ALP-S—alkaline phosphatase in supernatants, ALP-L—alkaline phosphatase in cell lysates, COLL-S—collagen levels in supernatants, COLL-l-—collagen levels in cell lysates).

Collagen levels quantified in supernatants, as an indicator of collagen secretion into the extracellular space, revealed a significant increase in MSCs in response to JR extract and EA and in mature osteoblasts treated with EA. The cellular response to JR, EA and CAT treatments in terms of ALP activity was primarily dependent on the stage of cellular differentiation. JR extract and especially EA increased ALP activity in extracellular and intracellular compartments in MSC cultures after 10 days. The highest increase in ALP activity was observed in mature osteoblasts (O10), and its activity was inhibited in pre-osteoblasts (O6) with all treatments after 10 days. As can be seen in the schematic diagram in Figure 19, JR extract did not have a significant effect in inducing mineralization, but did have a significant effect on the construction of the extracellular protein matrix (collagen, OPN and OC). This behavior can be explained by higher bone remodeling, in which average tissue mineralization is reduced and cellular heterogeneity is increased. This fact is described by Allen M.R et al. (2019), who state that older, more mineralized bones are replaced by newer bone tissue, which has lower mineralization [36].

The significance of the values obtained for NF-kB in our experiments is given by the type of treatment applied. EA and JR did not influence NF-kB levels. However, CAT, which proved to be slightly cytotoxic at the doses used in this study, induced significant decreases in all three cell lines, with MSCs being more sensitive.

NF-kB is a transcription factor implicated in inflammation, apoptosis and cellular degradation processes. Inflammation induced by TNF-β on MSCs induced suppression of osteogenesis with downregulation of bone-specific matrix β1-integrin and Runx2 genes and upregulation of NF-κB phosphorylation. p105 is an inhibitory protein that maintains NF-κB heterodimers, p50 and p65, in the cytoplasm. NF-κB canonical activation by cytokines (such as TNF-α and RANKL) results in p105 phosphorylation, leading to its degradation and allowing for nuclear translocation of NF-κB. Therefore, it highlights the formation and activity of osteoclasts induced by inflammation in bone diseases [37,38]. In a previous study, resveratrol reversed TNF-β through NF-κB downregulation by activating Sirt1 (Sirtuin 1) and Runx2, thus demonstrating MSC differentiation into osteoblasts [39]. Hess et al. in a study on hMSCs treated with TNF-α showed activation of NF-kB, which in turn enhanced BMP-2 and ALP expression concomitant with increased matrix mineralization. A decrease in NF-kB signaling did not result in a decrease in matrix mineralization [14].

Caro et al. demonstrated in various in vitro models of oxidative stress at the hepatic level that catechin can lead to an antioxidant or pro-oxidant profile depending on various factors not well described [40]. Such findings may guide us towards an interpretation of our results, in which free CAT, although in concentrations similar to those detected in JR extract, did not have similar effects on osteogenic differentiation as those of JR and EA treatments. NRF2 studied by immunocytochemistry showed an increase in expression after treatments with JR and EA, especially in younger cells, MSCs and O6 cells. Mature osteoblasts O10 expressed higher positivity in control samples and CAT-treated cells. An explanation for this increase in NRF2 expression is given by a different mechanism independent of the oxidative stress response by phytochemical compounds such as curcumin, EGCG and resveratrol. It was proposed that the NRF2 signaling pathway can be activated by phytochemicals by inducing phosphorylation of NRF2 via activation of upstream protein kinases and/or through direct interaction with Keap1 cysteine thiols. Subsequent synthesis of cytoprotective enzymes can suppress oxidative stress and the inflammatory response [41].

Phytotherapy approaches have been studied particularly in ovariectomized animal models for the induction of osteoporosis. There are numerous such studies in the literature, from which a few have been selected in this paper. *Piper sarmentosum* extract, a Chinese plant from the barberry family, was used for fractures of osteoporotic status and improved fracture healing [42]. Another plant originally from North America belonging to the family *Ranuculaceae*, *Cimicifuga racemosa*, is currently used in complementary and alternative therapies to improve postmenopausal syndrome [43]. *Cimicifuga racemosa* rhizomes contain some suggestive phytochemicals such as triterpene glycosides, phenols, flavonoids and alkaloids, ferulic acid, caffeic acid and isoflavones phytoestrogen (formononetin), the latter being responsible for the estrogenic activity [44,45].

Plant extracts obtained from *Eucommiae cortex*, *Salvia miltiorrhiza* and *Curcuma longa* have been successfully applied in the treatment of fractures because of their strong anti-inflammatory effects. These properties are not due to a single component, but rather a complex mixture of bioactive compounds with synergistic action on the bone microstructure [2]. QEP, a traditional Chinese medicine formula composed of *E. ulmoides* Oliv., *P. corylifolia* L., *A. sativum* L. and *J. regia* L., has shown beneficial effects on osteoporosis in ovariectomized rats and in vitro on human osteoblastic cell cultures hFOB 1.19. Alizarin red quantification also showed mineralization of hFOB 1.19 cells, and ALP determination supported osteogenic differentiation [46]. Pang et al. promoted osteoblast differentiation of human bone marrow-derived mesenchymal stromal cells (hBMSCs) by using JR leaf extract, indicating improved expression of pro-osteogenic biomarkers such as ALP, OCN or OPG. However, the extract did not present a significant effect on cell proliferation, while ALP determination revealed that walnut leaf extract improved osteogenic activity depending on the dose [24].

Papoutsi et al. showed that EA and methanolic JR extract significantly induced nodule formation on KS483 osteoblasts, promoting mineralization, meanwhile also providing a high anti-atherogenic potential. The study was also conducted on human aorta endothelial cells. Ellagic acid at a low concentration induced ossification, while JR methanolic extract also activated the mineralized nodules’ formation [47]. In our study in an osteogenic environment, the treatments with JR and EA, as well as the control group, initiated the formation of osteogenic nodules, while the treatment with CAT had no mineralization effect. Similar findings were reported by Dong et al. In this in vitro study, EA promoted the osteoblastic differentiation of BMSCs and induced osteogenesis in ovariectomized mice by increasing bone markers such as ALP, collagen 1 alpha 1, osteopontin and osteocalcin. On the seventh day of treatment, the ALP and alizarin red staining results indicated significant osteogenic differentiation in the EA groups compared to the control [48].

In our previous study, several polyphenolic compounds were identified in JR leaf extract such as ellagic acid, caffeic acid, ferulic acid, quercetin, resveratrol and catechin [19,25]. Each biocompound in JR extract can exert its antioxidative and anti-inflammatory action in a synergistic manner. *Juglans regia L.* extract compared to ellagic acid on bone neoformation in rats has also been studied individually in relation to its effect on osteogenesis (RANKL and hydroxyproline serum levels), oxidative stress (MDA levels, and SOD and CAT activities) and the anti-inflammatory effect (blood levels of TNFα and IL-6) in our previous in vivo study. The conclusion of the study was that oxidative stress was diminished in the presence of JR extract (MDA levels were lowest in the JR group), while the proinflammatory mediators were not significantly modified. Also, regarding bone regeneration and reabsorption markers, RANKL levels were low in the JR-treated group, and hydroxyproline concentration was not significantly improved [25].

Santos et al. in an in vitro study on osteoblast-like cell cultures treated with caffeic acid phenethyl ester showed that caffeic acid at low concentrations induced an increase in Rux2, Alp and SPP1 gene expression after 5 days of treatment and an enhancement in mineralization after 14 days [49]. The effects of caffeic acid were also presented in an in vivo study using a rat cranial defect model, demonstrating significantly improved bone defect healing [50]. Lotfi et al. showed that quercetin expressed outstanding osteogenic activity, anti-inflammatory effects and antioxidant capacity, all of which can be used for improving the bone regeneration process [18]. Liang et al. proved the potential of ferulic acid in promoting the proliferation and differentiation of skeletal stem cells after irradiation [51].

Catechins, an important class of natural polyphenolic phytochemicals, have multiple therapeutics effects due to their osteoprotective, antioxidant, and anti-inflammatory properties. Catechin has shown a stimulatory effect on osteoblast growth in an in vitro study on osteoblastic MC3T3-E1 cells due to inhibition of TNF-α and IL-6, while increasing cell viability and ALP activity [52].

Epigallocatechin gallate was demonstrated to augment calcium and phosphate levels and to reduce IL-6, TNF and RANKL. EGCG showed biocompatible and osteoinductive properties; it also stimulated the expression of osteogenic growth factors and increased osteocalcin and ALP levels [53]. In another study conducted on osteoblast-like MC3T3-E1 cells, Kawabata et al. showed that EGCG repressed the effect of insulin-like growth factor-I in inducing the migration of osteoblasts [54]. One beneficial green tea compound is epicatechin gallate (ECG), which in a study on C3H10T1/2 cells and hMSCs stimulated the differentiation of osteoblasts [55]. ECG showed protective effects on MC3T3-E1 cells against cadmium-induced osteoblast apoptosis [56].

All of these studies demonstrate the complex effects induced by each of these bio-compounds, mainly with anti-inflammatory, antioxidative, osteoinductive and protective activity. The presence of these compounds in adequate proportions in the JR leaf extract allows for their synergistic action, thus explaining the differences in cellular response compared to EA and CAT treatments.

Our study presents several limitations in the interpretation of the results, including the use of a single donor and the assessment of indirect biomarkers of oxidative stress, which only partially reflect the effects of reactive oxygen species. Interindividual variability could influence cellular responses to treatment, an aspect that should be considered when interpreting the data. Another limitation is using a single concentration for each treatment in differentiation experiments. Nevertheless, we consider the obtained results to be a first, using JR extract, bringing new insights that support its osteoregenerative potential, as evaluated on multiple cell lines. Thus, a natural product such as JR extract, with its complex phytochemical composition, may open promising perspectives for the development of new phytotherapeutic solutions with clinical applicability. In future research, we aim to use a more robust experimental design with larger sample sizes to validate the current findings.

## 5. Conclusions

The complex process of bone regeneration is influenced by both cells (mesenchymal stem cells and osteoblasts) and the presence of osteoregenerative factors. In vitro studies performed on cells in different stages of development, such as adult mesenchymal stem cells, pre-osteoblasts and late-stage osteoblasts, showed that the cells responded differently to treatments with *Juglans regia* L. extract in comparison with EA and CAT. The obtained results highlight the protective effects and osteogenic differentiation generated by JR extract in a manner comparable to EA treatment, while a cytotoxic effect and increased sensitivity was observed under CAT administration. Our study provides new insights into understanding the function of JR leaves and presents new potential treatment options for osteoporosis.

## Data Availability

The original contributions presented in this study are included in the article/Supplementary Material. Further inquiries can be directed to the corresponding authors.

## Supplementary Materials

The following supporting information can be downloaded at: https://www.mdpi.com/article/10.3390/jfb16070268/s1.
