# Supplementary material for: Investigating the Osteoregenerative Properties of Juglans regia L. Extract on Mesenchymal Stem Cells and Osteoblasts Through Evaluation of Bone Markers: A Pilot Study
Source: J Funct Biomater. 2025 Jul 21;16(7):268. doi: 10.3390/jfb16070268 (PMC12295196; doi:10.3390/jfb16070268)
Supplement: Supplementary file 1 [file jfb-16-00268-s001.zip › jfb-3649708-supplementary.pdf]

Quantification of malondialdehyde (MDA) through fluorescence

Measuring lipid peroxides and aldehydes is the most used method for detecting oxidative stress. The quantification of lipid peroxidation products is based on the determination of the colored compound resulting from the reaction of malondialdehyde produced from the peroxidation reaction of lipid structures from different macromolecular compounds with 2-thiobarbituric acid. Malondialdehyde resulting from this peroxidation reaction forms a fluorescent adduct with thiobarbituric acid that can be detected spectrofluorimetric. For the determination, the plasma sample or tissue homogenate is boiled for one hour with a 10 mM 2-thiobarbituric acid solution in 75 mM K_2HPO_4 at pH3. After sudden cooling, the reaction product is extracted into n-butanol. Its concentration is determined in the organic phase after its separation by centrifugation. The emission intensity is measured at 534nm with a Perkin Elmer spectrofluorimeter using a synchronous fluorescence technique at a wavelength difference between excitation and emission ($\Delta\lambda$) of 14nm. The malondialdehyde concentration is established based on a calibration curve made with known concentrations of MDA processed in the same way. Concentration values are expressed in nmol mL^{-1}. In our experiments cell lysates obtained after culturing young osteoclasts (O6) for 10 days and performing three treatments with JR, EA and CAT were evaluated for MDA levels using this method.

Catalase activity

Catalase is one of the enzymes involved in protection against oxidative stress, whose action involves the decomposition of hydrogen peroxide (H_2O_2) into water and oxygen. The method of determining catalase activity consists in following the change in absorbance of a 10 mM H_2O_2 solution in 0.05 M potassium phosphate buffer (pH 7.4) at 240 nm. One unit of activity is arbitrarily defined as the amount of enzyme that induces a reduction in absorbance of 0.43 at 25°C for 3 minutes. The activity is expressed in U mg^{-1} protein calculated according to the following formula resulting from the working conditions: catalase activity = $A_{240}/0.43x0.02$ (mg mL^{-1}) where A_{240} is the absorbance at 240 nm. Catalase activity was measured from O6 cell lysates, after 10 day of culture in OS medium and application of three treatments with JR, EA and CAT.

Evaluation of antioxidative profile

The levels of malondialdehyde and catalase activity was quantified in the O6 cell lysates after 10 days cultures with the application of three treatments with JR, EA and CAT. Although there are some numerical differences between the determinations with increased levels of MDA for JR and CAT, the statistical analysis performed with one-way ANOVA did not detect significant statistical differences. (Figure A)

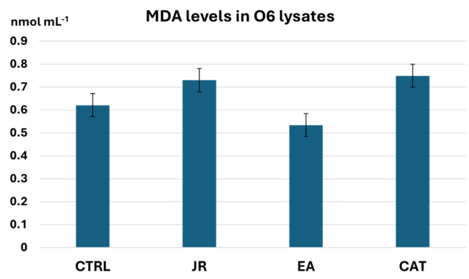

Figure A. Graphic representation of MDA (nmol mL^{-1}) evaluated from cell lysate samples of O6 cell cultures in osteogenic medium and 3 treatments of JR, EA and CAT, samples were collected at 10 days.

Catalase activity was higher after treatment with EA and JR, but statistical analysis did not identify significant differences. (Figure B)

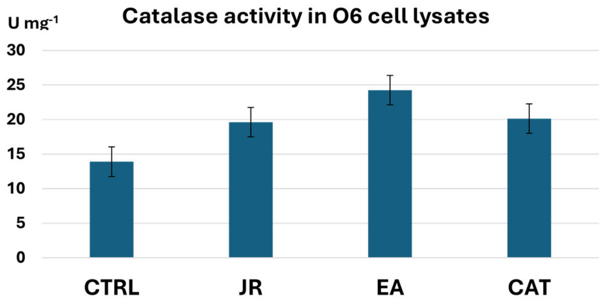

Figure B. Graphic representation of MDA (nmol mL^{-1}) evaluated from cell lysate samples of O6 cell cultures in osteogenic medium and 3 treatments of JR, EA and CAT, samples were collected at 10 days.
